# Bumetanide Exposure Association with Alzheimer’s Disease Risk

**DOI:** 10.21203/rs.3.rs-2574215/v1

**Published:** 2023-02-28

**Authors:** Anna Graber-Naidich, Justin Lee, Kyan Younes, Michael D. Greicius, Yann Le Guen, Zihuai He

**Affiliations:** Stanford University; Stanford University; Stanford University; Stanford University; Stanford University; Stanford University

**Keywords:** Alzheimer’s disease, bumetanide, electronic health record informatics, furosemide

## Abstract

**Background:**

To investigate whether exposure history to two common loop diuretics affects the risk of developing Alzheimer’s disease (AD) after accounting for socioeconomic status and congestive heart failure.

**Methods:**

Individuals exposed to bumetanide or furosemide were identified in the Stanford University electronic health record using the deidentified Observational Medical Outcomes Partnership platform. We matched the AD case cohort to a control cohort (1:20 case:control) on gender, race, ethnicity, hypertension and controlled for variables that could potentially be collinear with bumetanide exposure and/or AD diagnosis. Among individuals older than 65 years, 5,839 AD cases and 116,103 matched controls were included. A total of 1,759 patients (54 cases, 1,705 controls) were exposed to bumetanide.

**Results:**

After adjusting for socioeconomic status and other confounders, bumetanide exposure was significantly associated with reduced AD risk (odds ratio = 0.50; 95% confidence interval, 0.37-0.68; p = 9.9×10^−6^), while the most common loop diuretics, furosemide, was not associated with AD risk.

**Conclusion:**

Our study replicates in an independent sample that history of bumetanide exposure is associated with reduced risk of AD and emphasizes that this association is not confounded by difference in socioeconomic status, which was an important caveat given the cost difference between bumetanide and furosemide.

## Introduction

Medical systems generate massive amounts of Electronic Health Record (EHR) data and researchers have analyzed these data to derive new insights and improve healthcare^1,2^. Stanford University has established a novel and secure data platform: Observational Medical Outcomes Partnership (OMOP) Common Data Model (CDM). Alzheimer’s disease (AD) is well-suited for analysis with OMOP given its multifaceted complexity, prevalence, and the multitude of small size studies that claim benefit for certain interventions.

AD is a neurodegenerative disorder of uncertain cause and pathogenesis. In the United States, as many as 1 in 9 people (10.7%) older than 65 have AD^3^. Recently, repurposing bumetanide as an AD medication was proposed based on data that showed bumetanide “reversed” APOE genotype-dependent transcriptomic signatures in mouse and cell culture models.^4^ This finding was investigated in two EHR-based cohorts demonstrating that in individuals over 65 years of age, bumetanide exposure was associated with lower AD prevalence^4^. This finding warrants further validation as bumetanide is more expensive than the commonly prescribed loop diuretic (furosemide) and thus potential socioeconomic status (SES) confounding such as insurance coverage needs to be investigated. Both furosemide and bumetanide are indicated, and often interchangeably used, for patients with hypertension, congestive heart failure (CHF), and kidney disease. In this study, using Stanford’s EHR data, we sought to replicate the bumetanide findings in an independent dataset accounting for SES, hypertension, and CHF.

A total of 1,759 patients (54 cases, 1,705 controls) were exposed to bumetanide during any of their visits (Table 1). For the full matched cohort, the bumetanide prevalence among AD cases was 0.92% compared to 1.47% in the controls. The unadjusted odds ratio (OR) for AD diagnosis among bumetanide exposed was 0.63 (95% CI, 0.48-0.82; p = 7.6×10^−4^, Table 2).

We had some missingness in our data for the SES variables – insurance information was available for 94.0% of cases and 84.5% of controls. In addition, for the zip code data informing the median income component, we focused on patients from California, and due to deidentification reasons the data were only available for 76.8% of cases and 74.1% of controls. Since SES estimates were not easily imputable from our data, a sensitivity analysis was performed as a complete case analysis. In this sensitivity analysis we restricted the cohort to patients with complete insurance and median income data (N=77,688) and repeated the unadjusted analysis prior to fitting a multivariable model adjusted for CHF, insurance, and median income. In the complete case restricted cohort, 45/4238 (1.06%) AD cases and 1,321/7,3450 (1.8%) matched controls were exposed to bumetanide. The unadjusted OR remained similar to the one in our primary analysis (OR=0.59; 95% CI, 0.43-0.79; p = 4.5×10^−4^, Table 2). After adjusting for CHF, insurance, and median income the estimated OR for AD diagnosis among bumetanide exposed was 0.50 (95% CI, 0.37-0.68; p = 9.9×10^−6^, Table 2).

For the full matched cohort, the furosemide prevalence among AD cases was 18.8% compared to 17.2% in the controls. The unadjusted OR for AD diagnosis among furosemide exposed was 1.11 (95% CI, 1.03-1.18; p = .003, Table 2); however, this effect was not replicated when adjusting for CHF.

Most clinical trials in AD suffer from an inherent shortfall regarding primary prevention as they do not give insights on whether a compound reduces the incidence of AD since medications are tested after disease onset. Studying EHR using OMOP allows us to derive insight on possible primary prevention of AD^5^.

In an independent dataset, our results replicate the original study that found a protective effect of Bumetanide exposure on AD risk^4^. We further investigated whether this effect is generalizable to the more commonly used and less expensive medication in the same class and adjusted for potential confounding variables such as SES and CHF. In our study, despite their similar indications and mechanism of action, only bumetanide exposure associated with reduced future AD diagnosis. Notably, both bumetanide and furosemide seem to penetrate the blood brain barrier^6–9^. Potential explanations for these results include unique effects of bumetanide on the *APOE* genotype-dependent transcriptomic signature,^4^ bumetanide being a more potent medication than furosemide with a dose ratio of 1:40 mg, or potential protective molecular modulation of neuronal transmembrane chloride gradients by blocking NKCC1 in the central nervous system.^10^ A mechanism that led to its proposed investigations to treat autism,^11^ schizophrenia,^12^ and epilepsy^12,13^.

Our OMOP EHR dataset analysis demonstrated potential association between past bumetanide exposure and AD onset. This effect remained significant even after correcting for SES and CHF indicating that the results are not driven by differences in SES or severity of cardiac disease.

These results should be treated cautiously since they are based on retrospective data. Bumetanide is a potent loop diuretic which, if given in excessive amounts, can lead to a profound diuresis with water and electrolyte depletion which is particularly problematic in the elderly population. Additionally, insurance and income were modeled through proxies available in OMOP and may not fully account for difference in SES. Last, additional functional studies are warranted to investigate the biological mechanism through which bumetanide exposure is associated with reduced AD risk. The current findings do not support the use of bumetanide for the prevention or treatment of AD. There is a need for prospective, randomized, double-blinded, placebo-controlled clinical trials to confirm the findings in patients without comorbidities and determine the lowest effective dose that may reduce the risk of AD without causing intolerable side effects. Given the lack of effect of furosemide and its similar side effect profile, one could also consider using it as an active placebo to increase the efficacy of double blinding.

## Methods

Stanford’s deidentified OMOP instance hosts multi-factor and multi-modal data, including Stanford’s structured clinical data, clinical notes, meta-data on clinical notes, extracted concepts from clinical notes using Natural Language Processing and other approaches, and radiological images. Participants or their caregivers provided written informed consent for their data to be stored in OMOP. The current study protocol was granted an exemption by the Stanford University institutional review board because the analyses were carried out on deidentified data; therefore, additional informed consent was not required. **All experimental protocols were approved by were approved** by the Stanford University institutional review board.

We have curated the OMOP data for 656,683 patients over age 65 at their last known visit. We focused on 5,872 patients with AD defined by ICD9 and ICD10 codes (ICD10: G30.1, G30.8, G30.9 and ICD9:331.0). We matched individual AD patients with up to 20 controls on age and exact match of sex, race, ethnicity, and hypertension with the R package *optmatch*^14^. We excluded 937 matched controls that had an age difference greater than 5 years and 373 matched controls that belonged to strata that had fewer than 15 controls per case resulting in 5,839 AD cases and 116,103 matched controls (Table 1).

### Statistical analysis

We scanned the data using the medication orders or medical history variable tables to identify those who had been exposed to bumetanide. We included any type of exposure to the drug, specifically oral or IV exposures for any duration of time. Additionally, as post-hoc sensitivity analyses, we controlled for variables that could potentially be collinear with bumetanide exposure and/or AD diagnosis including diagnosis of CHF (defined by the ICD10 of I11, I13, I50), insurance type, and median income (defined by the patient’s recorded zipcode, derived from publicly available data form the United States Census Bureau)^5^ and explored the relationship of AD with the other commonly used loop diuretic, furosemide. We calculated the prevalence of exposure to bumetanide in cases compared to the non-AD control group using a χ2 test. Statistical analyses were performed using R (version 3.6.3).

## Figures and Tables

**Table 1.** Demographics of participants by diagnosis status. Each AD patient was matched with up to 20 controls on age and exact match of sex, race, ethnicity, and hypertension.

	AD Case	Matched Control	SMD
**n**	5,839	116,103	
**Age at last visit (median [IQR])**	84.00 [79.00, 88.00]	83.00 [79.00, 87.00]	0.058
**Gender = MALE (%)**	2256 (38.6)	45134 (38.9)	0.005
**Race (%)**			0.013
**White**	3636 (62.3)	72719 (62.6)	
**Asian**	835 (14.3)	16633 (14.3)	
**Black or African American**	262 (4.5)	4961 (4.3)	
**Native Hawaiian or Other Pacific Islander**	38 (0.7)	700 (0.6)	
**American Indian or Alaska Native**	4 (0.1)	80 (0.1)	
**Patient Refused**	65 (1.1)	1271 (1.1)	
**Other**	755 (12.9)	14869 (12.8)	
**Unknown**	205 (3.5)	4100 (3.5)	
**No matching concept**	39 (0.7)	770 (0.7)	
**Ethnicity (%)**			0.001
**Not Hispanic or Latino**	5022 (86.0)	99824 (86.0)	
**Hispanic or Latino**	437 (7.5)	8717 (7.5)	
**No matching concept**	380 (6.5)	7562 (6.5)	
**Hypertension (%)**	3667 (62.8)	72663 (62.6)	0.004
**Congestive heart failure (%)**	414 (7.1)	5627 (4.8)	0.095

**Table 2.** Odds ratio for AD diagnosis among bumetanide and furosemide exposed participants. Individuals were over the age of 65 years, and odds ratio were adjusted for age, sex, race, ethnicity, hypertension and with and without adjusting CHF and SES (insurance, and median income). AD: Alzheimer’s Disease, CHF: Congestive Heart Failure, SES: Socioeconomic Status. OR: Odds Ratio.

Analysis	OR (95% CI)	p-value
Unadjusted matched cohort bumetanide exposure	0.63 (0.48, 0.82)	7.6×10^−4^
CHF adjusted matched cohort bumetanide exposure	0.52 (0.39, 0.68)	3.1×10^−6^
Unadjusted matched cohort furosemide exposure	1.11 (1.03, 1.18)	.003
CHF adjusted matched cohort furosemide exposure	1.02 (0.94, 1.09)	.70
Unadjusted complete case cohort bumetanide exposure	0.59 (0.43, 0.79)	4.5×10^−4^
CHF adjusted complete case cohort bumetanide exposure	0.49 (0.36, 0.67)	5.0×10^−6^
CHF, SES adjusted complete case cohort bumetanide exposure	0.50 (0.37, 0.68)	9.9×10^−6^

## Data Availability

This research used data or services provided by STARR, “STAnford medicine Research data Repository,” a clinical data warehouse containing live Epic data from Stanford Health Care (SHC), the Stanford Children’s Hospital (SCH), the University Healthcare Alliance (UHA) and Packard Children’s Health Alliance (PCHA) clinics and other auxiliary data from Hospital applications such as radiology PACS. STARR platform is developed and operated by Stanford Medicine Research IT team and is made possible by Stanford School of Medicine Research Office.

## Abbreviations

AD: Alzheimer’s Disease
EHR: Electronic Health Record
CDM: Common Data Model
OHDSI: Observational Health Data Sciences and Informatics
OMOP: Observational Medical Outcomes Partnership
